# Personalized versus standardized dosing strategies for the treatment of childhood amblyopia: study protocol for a randomized controlled trial

**DOI:** 10.1186/s13063-015-0711-4

**Published:** 2015-04-25

**Authors:** Merrick J Moseley, Michael P Wallace, David A Stephens, Alistair R Fielder, Laura C Smith, Catherine E Stewart, RODS (Randomized Occlusion Dosing Strategies) Study Cooperative

**Affiliations:** Division of Optometry and Visual Science, City University, London, EC1V 0HB UK; Department of Epidemiology, Biostatistics and Occupational Health, McGill University, Montreal, Canada; Department of Mathematics and Statistics, McGill University, Montreal, Canada

**Keywords:** amblyopia, child, occlusion therapy, personalized dosing strategy, randomized clinical trial, standardized dosing strategy, total effective dose, visual acuity

## Abstract

**Background:**

Amblyopia is the commonest visual disorder of childhood in Western societies, affecting, predominantly, spatial visual function. Treatment typically requires a period of refractive correction (‘optical treatment’) followed by occlusion: covering the nonamblyopic eye with a fabric patch for varying daily durations. Recent studies have provided insight into the optimal amount of patching (‘dose’), leading to the adoption of standardized dosing strategies, which, though an advance on previous *ad-hoc* regimens, take little account of individual patient characteristics. This trial compares the effectiveness of a standardized dosing strategy (that is, a fixed daily occlusion dose based on disease severity) with a personalized dosing strategy (derived from known treatment dose-response functions), in which an initially prescribed occlusion dose is modulated, in a systematic manner, dependent on treatment compliance.

**Methods/design:**

A total of 120 children aged between 3 and 8 years of age diagnosed with amblyopia in association with either anisometropia or strabismus, or both, will be randomized to receive either a standardized or a personalized occlusion dose regimen. To avoid confounding by the known benefits of refractive correction, participants will not be randomized until they have completed an optical treatment phase. The primary study objective is to determine whether, at trial endpoint, participants receiving a personalized dosing strategy require fewer hours of occlusion than those in receipt of a standardized dosing strategy. Secondary objectives are to quantify the relationship between observed changes in visual acuity (logMAR, logarithm of the Minimum Angle of Resolution) with age, amblyopia type, and severity of amblyopic visual acuity deficit.

**Discussion:**

This is the first randomized controlled trial of occlusion therapy for amblyopia to compare a treatment arm representative of current best practice with an arm representative of an entirely novel treatment regimen based on statistical modelling of previous trial outcome data. Should the personalized dosing strategy demonstrate superiority over the standardized dosing strategy, then its adoption into routine practice could bring practical benefits in reducing the duration of treatment needed to achieve an optimal outcome.

**Trial registration:**

ISRCTN ISRCTN12292232.

## Background

Amblyopia is the commonest visual disorder of childhood in developed nations, with an estimated prevalence of 1.6 to 3.5% [1]. Principally manifesting as a deficit of visual acuity in one eye, it is most commonly found in association with squint or anisometropia (unequal refractive error between eyes), or both. Historically, a diverse range of physical and pharmacological treatments have been prescribed [2], and although novel treatments continue to emerge (for example, those involving visual stimulation) [3-5], occlusion of the nonamblyopic eye, most commonly by an adhesive fabric patch, remains the treatment of preference [6].

For the most part of the twentieth century, the efficacy of occlusion went unquestioned and it was only with the championing of evidence-based medicine in the 1990s [7] that its effectiveness became the subject of systematic investigation [2,8]. One strand of observational research has sought to determine mathematically the relationship (‘dose response’) between the amount of patching and any subsequent change in visual acuity. Such studies were facilitated by the introduction of the occlusion dose monitor by Fielder and colleagues in 1994 [9,10]. This device permitted, for the first time, objective records of patching episodes to be obtained from children undergoing treatment. Using this device, two principal studies, MOTAS and ROTAS [11,12], have provided insight into the dose-response relationships of occlusion therapy and how these vary as a function of amblyopia type, age of patient, and severity of the condition. The findings of these studies have largely been confirmed by traditional randomized trials [13] and have helped to identify optimal treatment regimens for use in clinical practice.

A practical consequence of this research has been a trend away from *ad-hoc* patching regimens (anything from a few minutes per day up to all waking hours) towards a more standardized approach (now gradually being incorporated into practice guidelines [14]). Thus, it is now possible to elicit consensus among expert practitioners as to what presently constitutes best practice, that is, in the amount of patching that should be prescribed to an initially presenting amblyopic child. In this Randomized Occlusion Dosing Strategies (RODS) trial, the occlusion regimens prescribed in one of the treatment arms (the standardized dosing strategy) represents the current prevailing clinical opinion of a representative group of UK orthoptists.

The rationale behind RODS is the notion that further refinements in prescribing may yield improvements in outcome. By examining the dose-response relationships determined for the amblyopic children taking part in MOTAS [11] and ROTAS [12], we have generated a statistical model that allows us to estimate, on the basis of the severity of the initially presenting amblyopia, the age of the patient, and the type of amblyopia, the amount (total hours) of occlusion required to treat any given amblyopic child. This we term the ‘total effective dose’, which for any given treatment period can be converted into a daily dose-rate (hours per day) and subsequently increased or decreased depending on compliance. We term this approach a ‘personalized dosing strategy’.

In summary, the protocol described herein has been designed to test the hypothesis that superior clinical outcomes will be obtained with a personalized dosing strategy compared with a standardized dosing strategy (in which all patients receive an occlusion regimen based upon current best practice).

## Methods/design

### Objectives of the study

The primary study objective is to determine whether study participants in receipt of a personalized dosing strategy reach trial endpoint (optimal visual acuity) in a shorter period of time, and with reduced variance, than those in receipt of a standardized dosing strategy.

The secondary study objectives are:

1. To determine the relationship between observed changes in logMAR visual acuity as a function of:
  Objectively monitored occlusion dose,
  Type of amblyopia,
  Age of participant.
2. To determine whether compliance differs between standardized and personalized dosing strategy treatment arms.

### Eligibility criteria

#### Inclusion criteria

- Male or female,
- Aged 3 to 8 years inclusive,
- Visual acuity of 0.10 logMAR or higher (poorer acuity) in the worst (amblyopic) eye,
- Interocular difference in acuity of at least 0.20 logMAR,
- Presence of anisometropia or strabismus (or both),
- Knowledge of the extent of spectacle wear (if any) prior to trial entry,
- Parent or guardian willing and able to give informed consent for participation in the study,
- Cognitive, motor and verbal skills of sufficient maturity to undergo visual acuity testing with letter optotypes.

#### Exclusion criteria

- Comorbid ocular disease (including amblyopia associated with form deprivation),
- Prior occlusion therapy for amblyopia,
- Evidence of learning difficulties or developmental delay.

### Study design

**Figure 1:**
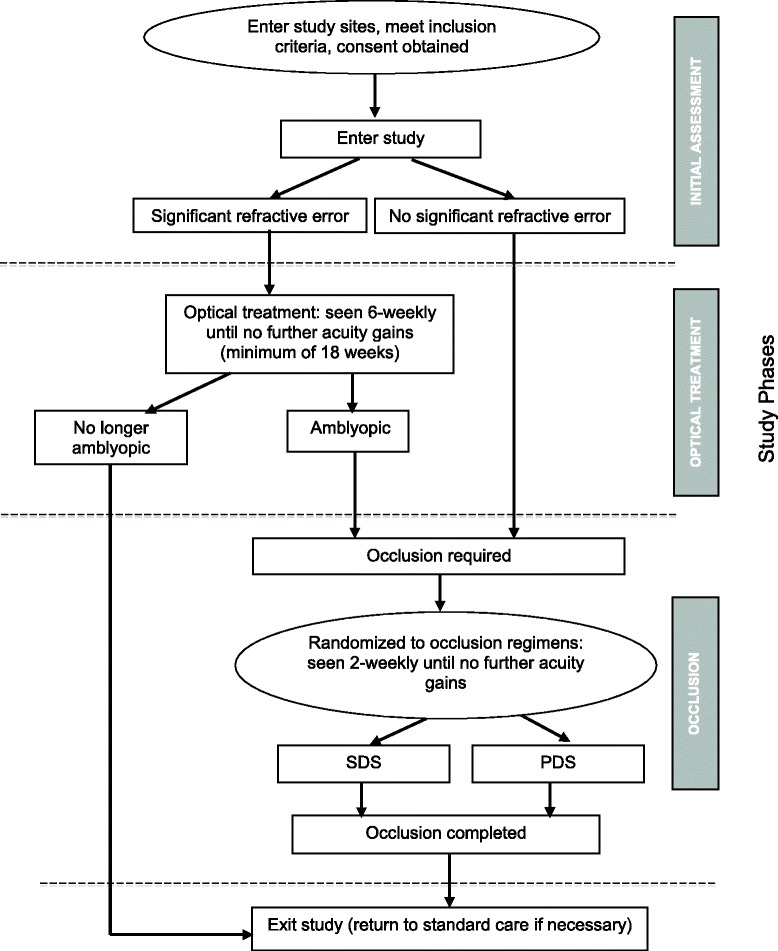
Study participant flow.

The study is a randomized, parallel group, unmasked design, as depicted in Figure 1. Each study arm comprises three sequential phases: initial assessment, optical treatment (common to each respective study arm) and occlusion (either standardized or personalized dosing strategy in respective study arms).

### Dosing strategies

#### Standardized dosing strategy

In the standardized dosing strategy arm, participants are prescribed one of three dosing regimens dependent on their visual acuity at the end of the optical treatment phase. These dosing regimens were the consensus outcome, initially of formal face-to-face discussion, between eight senior orthoptists identified by membership of the British and Irish Orthoptic Society, and drawn from geographically diverse areas of the United Kingdom. All experts had a minimum of 10 years’ clinical experience. Discussion focused on the management of six clinical scenarios representative of cases meeting the RODS eligibility criteria. The experts’ views of how each scenario should be managed were appraised by the panel convener (CES) and a series of dosing regimens was derived. These regimens were subsequently reviewed by the panel and their suitability unanimously confirmed by all members as follows:

- Mild amblyopia (<0.4 logMAR): 2 hours per day,
- Moderate amblyopia (0.4 to <0.8 logMAR): 3 hours per day,
- Severe amblyopia (≥0.8 logMAR): 5 hours per day.

#### Personalized dosing strategy

In the personalized dosing strategy arm, participants are prescribed an initial occlusion dose determined by their age, type, and severity of amblyopia, based upon dose-response functions derived from previous studies involving participants of near-identical clinical and demographic status to those enrolled in the present trial [11,12]. As participants in this arm progress through the occlusion phase, the prescribed occlusion dose is modified at each clinic visit, depending on treatment compliance, and is predicated on the notion of a total effective dose: the minimum amount of occlusion that should result in an optimal visual outcome.

The rationale underpinning treatment by a personalized dosing strategy is an attempt to define a patient’s total occlusion dose, which can then be converted to a daily patching regimen on the presumption of a fixed treatment period. We make the assumption that every patient has some optimal visual outcome that they can hope to achieve, and that there exists some total effective dose of occlusion that must be received in order to achieve it. Furthermore, we assume that any dose received by a patient in excess of this total effective dose is ‘wasted’: it does not improve the patient’s visual acuity any further, as it is impossible to improve beyond the patient’s optimal visual outcome. For example, if a patient’s total effective dose is 200 hours, then that patient will have the same visual outcome after 200, 250, or even 1,000 hours of occlusion. However, the patient’s visual improvement would be limited if fewer than this 200 hours of occlusion were achieved. Employing mathematical notation, we can say that patient *i* has some optimal visual acuity *V*_i_^*^, which will be achieved if, and only if, patient *i* undertakes at least *D*_i_^*^ hours of occlusion. Our aim is to construct a model that predicts this *D*_i_^*^ based on an analysis of known treatment-response functions [11,12]. To constrain the complexity of the model (and in the absence of compelling evidence to the contrary) it is assumed that the total effective dose is dose-rate independent, that is, a single occlusion dose is of equivalent effectiveness to multiple individual doses that sum to the same total number of hours.

Predictions of *D*_i_^*^ were derived from a normal linear interval regression model with the total effective dose of some patient *i* with initial residual amblyopia *R*_*i*_ and age *A*_*i*_ estimated by the formula:

$$ {\mathrm{D}}_{\mathit{\mathsf{i}}}^{*}={\left(18.150+16.122{R}_i\hbox{--} 0135{A}_i\hbox{--} 0.547{T}_M\hbox{--} 8.028{T}_S\hbox{--} 12.176{R}_i{T}_M\hbox{--} 11.334{R}_i{T}_S+0.065{A}_i{T}_M+0.192{A}_i{T}_{\mathrm{S}}\right)}^2 $$

where *T*_M_ and *T*_S_ are indicator functions taking the value 1 if the patient has mixed or strabismic amblyopia, respectively, or 0 otherwise.

In practice, participants in the personalized dosing strategy arm have their total effective dose estimated on an assumed fixed follow-up period of 12 weeks, which results in manageable dose-rates. Therefore, an individual patient’s personalized daily patching regimen will be calculated by first estimating the total effective dose and then dividing this by the number of days left in the follow-up period. For reasons of practicality, we only prescribe daily patching episodes rounded up to the nearest 30 minutes. Dose-rate can then be updated at follow-up appointments based on how much occlusion has been objectively recorded. To avoid the prescription of subtherapeutic doses of occlusion, the model is constrained such that dose-rates less than 2 hours per day are never prescribed. Of course, patients will probably be treated for a period longer than 12 weeks, as the follow-up sessions will be continued until the visual acuity criteria for study exit have been met. Therefore, once a patient passes the tenth week of follow-up, all future daily prescription calculations will assume a 2-week follow-up period until treatment is completed.

To illustrate implementation, consider a hypothetical patient who enters occlusion aged 48 months with residual amblyopia of 0.5 logMAR and amblyopia associated with anisometropia. Using the formula derived from our model, the estimated total effective dose would be 389 hours. Over a 12-week follow-up period, this requires an average daily dose of 4.6 hours per day, and so we initially prescribe 5 hours of occlusion a day. At a follow-up appointment 2 weeks later, the patient has only undertaken 30 hours of occlusion (rather than the prescribed 70). The patient’s total effective dose reduces from 389 to 359 hours, but with only 10 weeks left of the initial follow-up period the patient must now average 5.1 hours of occlusion a day. Thus, our prescribed dose-rate increases to 5.5 hours a day.

### Recruitment sites

Potential study participants are initially identified from new patients attending orthoptic departments at Hillingdon Hospital, Hillingdon, UK and Princess Alexandra Hospital, Harlow, UK.

### Trial phases

#### Initial assessment phase

Attending patients undergo a full orthoptic and ophthalmic examination, including cycloplegic refraction. Where clinically significant refractive error is found, spectacles are prescribed (full corrections for astigmatic, anisometropic myopic and mild or moderate hypermetropic (+2.00 to +5.00) and within 2 dioptres of the full correction for high hypermetropic errors). Clinically significant refractive error is defined as:

- ≥1.50 dioptres sphere bilateral hypermetropia,
- ≥1.50 dioptres sphere bilateral myopia,
- ≥0.75 dioptres cylinder bilateral astigmatism,
- All astigmatism in combination with hypermetropia,
- ≥1.00 dioptres sphere anisometropia.

The parents or guardians of those children meeting the study’s eligibility criteria are provided with an information sheet and, subject to verbal consent, invited to attend an appointment (start of trial) with a research orthoptist (CES or LCS). They are asked to bring with them (but not to have their child previously wear) any spectacles dispensed as an outcome of the current consultation.

#### Optical treatment phase

Written informed consent is sought from the parents or guardians of all potentially participating children at the outset of this phase. Except for those children not prescribed spectacles in the initial assessment phase (who progress directly to the occlusion phase), participants are instructed to wear their spectacles full time and to return for vision assessment at 6-weekly intervals from week 0 (onset of spectacle wear) until 18 weeks of optical treatment is complete (a period that allows for all significant improvement attributable to spectacle wear to have occurred [15]). If there is a significant improvement (≥0.1 logMAR) between weeks 12 and 18, the participants are asked to wear their spectacles for further 6-week periods until significant gains have ceased. If participants have worn spectacles for refractive correction prior to study entry, the number of weeks for which spectacles were previously worn is documented; a minimum of 18 weeks (in total) wear is required before progression to the next study phase. It is anticipated that around 20 to 25% of participants will, by the end of this phase, have obtained equal eye acuity and will leave the trial at this point.

As a safeguard, should significant deterioration in acuity occur (defined as loss of more than 0.20 logMAR from any previous testing), the child will undergo a repeat cycloplegic refraction and the prescription will be changed if there is a significant difference from the initial prescription, before recommencing in this study phase. If no discrepancy is observed, in the presence of sustained deterioration in vision, the child shall be withdrawn from the trial. If a pathological cause of the vision loss is suspected, the child is referred back to the ophthalmologist at the local site for continued clinical care.

#### Occlusion phase

On entering this phase, study participants are randomly assigned (stratified by geographic site and amblyopia type) to either the personalized or standardized dosing strategy arms of the trial using an online true random number generator [16].

Participants will have their visual acuity recorded every 2 weeks until the trial endpoint is reached. To provide a check against the unmasked nature of the study design, visual acuity is further recorded at the end of the occlusion phase by an examiner masked to the participants’ treatment arms.

### Occlusion dose monitoring

All study participants who progress to the occlusion phase have their prescribed patch wear (standardized or personalized dosing strategy) objectively monitored using an occlusion dose monitor. Briefly, this device monitors, and records electronically, episodes of patch-skin contact indicative of patch wear. At the scheduled clinic visits, the occlusion dose monitor is interrogated via a PC to provide a time history of patching episodes from which daily (‘dose-rate’) and accumulated (‘dose’) occlusion can be derived. A full description of the occlusion dose monitor’s construction and mode of operation has been reported elsewhere [9,10].

### Endpoint determination and sample size calculation

Participants complete the trial at that point in the occlusion phase at which equal visual acuity of each eye is achieved, or if vision has stabilized (defined as three inflexions in a plot of acuity against time or remaining unchanged over three consecutive visits). All children wear occlusion for a minimum of 6 weeks before discontinuing if no significant gains in visual acuity are observed. A sample size of 60 in each arm at 95% power and with a type I error rate of 0.05 corresponds to a Cohen’s *d* statistic of 0.605. The log-transformed time to best visual acuity across MOTAS [11] and ROTAS [12] was approximately normally distributed and had a mean (standard deviation) of 4.0133 (0.801). We could therefore expect to detect a difference of around 21 days in mean time to best visual acuity in our two groups.

### Outcomes and analyses

#### Primary outcome and its measurement

The primary outcome variable is logMAR visual acuity assessed on one or more of the following test charts: crowded logMAR, uncrowded logMAR, or modified ETDRS.

The crowded and uncrowded logMAR charts are viewed at a test distance of 3 m and comprise six letters, X V O H U Y, which were selected for their approximately equal legibility. There are four letters on each line, which provide a constant visual demand at each level of acuity. Each letter correctly read can be scored by interpolation as 0.025 logMAR with an acuity range of 0.800 to −0.300 logMAR (6/38 to 6/3), which can be extended to 1.4 to −0.300 logMAR by employing a log increment step reduction in viewing distance. Letter spacing is equal to 0.5 letter diameters. In the crowded chart, a black printed rectangle surrounds each row of letters at 0.5 letter diameters distance and of equivalent width to the letter height.

The modified ETDRS chart is viewed at a test distance of 4 m, giving an acuity range of 1.00 to −0.30 logMAR (6/60 to 6/3). This test range can be extended to 1.60 logMAR by reducing the viewing distance in log incremental steps. Each line comprises five letters and is scored on a letter-by-letter basis, providing interpolated acuity values of 0.02 logMAR resolution.

The chart used depends on the reading ability of the child and is generally age-dependent. The visual acuity test used in the initial assessment phase will be used throughout the trial period; however, if a child is judged able to progress to a more difficult test, this test will additionally be employed for the remainder of the trial. Visual acuity of the amblyopic eye is always recorded first; the rationale being that amblyopic eye visual acuity is the principal outcome variable and the visual acuity of this eye should be tested when the child is maximally alert and free of fatigue.

#### Analyses

The primary study objective of comparing time to best visual acuity between the two treatment groups, along with the secondary objective of comparing compliance, will be analyzed via standard *t* tests (to compare length of time and compliance) and Levene’s test (to compare variance in time). Suitable nonparametric alternatives will be used should concerns arise regarding the necessary distributional assumptions for these methods. The secondary objective to determine the relationship between changes in visual acuity and dose, amblyopia type, and age, will be investigated using standard regression modelling approaches.

### Ethical approval and conduct

Ethical approval for the trial was granted by The National Research Ethics Service Committee London, Bloomsbury, protocol No. PB-PG-0808-16087.

## Discussion

The trial protocol described herein aims to compare two means of prescribing occlusion therapy for childhood amblyopia. One trial arm (standardized dosing strategy) is considered representative of current best practice in the UK, the other (personalized dosing strategy), a regimen with an empirical basis formulated using a statistical modelling approach of documented patient response data. To the best of our knowledge, the personalized dosing strategy is an entirely novel approach to undertaking occlusion therapy, with a significant potential benefit of a reduction in the amount of treatment needed. This advantage goes beyond any anticipated economic benefit and bears directly on the unpleasantness of occlusion for children and the corresponding difficulty parents have in achieving compliance (objective estimates of occlusion received are less than half those prescribed [17]). Hence, the personalized dosing strategy is underpinned by the notion of a total effective dose: a prescription of occlusion that is sufficient and not excessive and is informed on a prospective basis by an individual’s compliance (actual dose received).

It is necessary to emphasize that the implementation of a personalized dosing strategy (either that developed for the present trial or future modifications of it) is entirely dependent on the use of occlusion dose monitoring. This important advance has hitherto been entirely confined to use within formalized research studies and is not used routinely in clinical practice despite the seemingly obvious advantages it brings in providing feedback to clinicians and parents. A demonstration of the superiority of the personalized dosing strategy approach would undoubtedly provide an incentive for its take-up in the clinic.

### Trial status

Participant enrolment commenced in October 2013 and the trial is scheduled to complete by December 2015.

## Abbreviations

logMAR: logarithm of the Minimum Angle of Resolution
RODS: Randomized Occlusion Dosing Strategies

## References

[1] Attebo K, Mitchell P, Cumming R, Smith W, Jolly N, Sparkes R (1998). Prevalence and causes of amblyopia in an adult population. Ophthalmology.

[2] Moseley M, Moseley M, Fielder A (2002). Amblyopia: treatment and evaluation. Amblyopia: a multidisciplinary approach.

[3] Hess RF, Thompson B (2013). New insights into amblyopia: binocular therapy and noninvasive brain stimulation. J AAPOS.

[4] Foss AJ, Gregson RM, MacKeith D, Herbison N, Ash IM, Cobb SV (2013). Evaluation and development of a novel binocular treatment (I-BiTTM) system using video clips and interactive games to improve vision in children with amblyopia (‘lazy eye’): study protocol for a randomised controlled trial. Trials.

[5] Levi DM (2012). Prentice Award Lecture 2011: removing the brakes on plasticity in the amblyopic brain. Optom Vis Sci.

[6] Holmes JM, Repka MX, Kraker RT, Clarke MP (2006). The treatment of amblyopia. Strabismus.

[7] Sackett DL, Rosenberg WM, Gray JA, Haynes RB, Richardson WS (1996). Evidence based medicine: what it is and what it isn’t. BMJ.

[8] Snowden SK, Stewart-Brown SL (1997). Preschool vision screening. Health Technol Assessment.

[9] Fielder AR, Auld R, Irwin M, Cocker KD, Jones HS, Moseley MJ (1994). Compliance monitoring in amblyopia therapy. Lancet.

[10] Fielder AR, Irwin M, Auld R, Cocker KD, Jones HS, Moseley MJ (1995). Compliance in amblyopia therapy: objective monitoring of occlusion. Br J Ophthalmol.

[11] Stewart CE, Moseley MJ, Stephens DA, Fielder AR (2004). Treatment dose-response in amblyopia therapy: the Monitored Occlusion Treatment of Amblyopia Study (MOTAS). Invest Ophthalmol Vis Sci.

[12] Stewart CE, Stephens DA, Fielder AR, Moseley MJ (2007). Objectively monitored patching regimens for treatment of amblyopia: randomised trial. BMJ.

[13] Gunton KB (2013). Advances in amblyopia: what have we learned from PEDIG trials?. Pediatrics.

[14] American Academy of Ophthalmology Pediatric Ophthalmology/Strabismus Panel. Preferred Practice Pattern® Guidelines. Amblyopia PPP – September 2012. San Francisco, CA, USA: American Academy of Ophthalmology; 2012. http://www.aao.org/preferred-practice-pattern/amblyopia-ppp--september-2012.

[15] Moseley MJ, Neufeld M, McCarry B, Charnock A, McNamara R, Rice T (2002). Remediation of refractive amblyopia by optical correction alone. Ophthalmic Physiol Opt.

[16] Random.org. www.random.org.

[17] Wallace MP, Stewart CE, Moseley MJ, Stephens DA, Fielder AR, Monitored Occlusion Treatment Amblyopia Study (MOTAS) Cooperatives (2013). Compliance with occlusion therapy for childhood amblyopia. Invest Ophthalmol Vis Sci.

